# Learning an Operant Conditioning Task Differentially Induces Gliogenesis in the Medial Prefrontal Cortex and Neurogenesis in the Hippocampus

**DOI:** 10.1371/journal.pone.0014713

**Published:** 2011-02-18

**Authors:** Maximiliano Rapanelli, Luciana Romina Frick, Bonifacio Silvano Zanutto

**Affiliations:** 1 Laboratorio de Biología del Comportamiento, IBYME-CONICET, Ciudad de Buenos Aires, Buenos Aires, Argentina; 2 Facultad de Ingenieria, Instituto de Ingeniería Biomedica, Universidad de Buenos Aires, Ciudad de Buenos Aires, Buenos Aires, Argentina; University of Queensland, Australia

## Abstract

Circuit modification associated with learning and memory involves multiple events, including the addition and remotion of newborn cells trough adulthood. Adult neurogenesis and gliogenesis were mainly described in models of voluntary exercise, enriched environments, spatial learning and memory task; nevertheless, it is unknown whether it is a common mechanism among different learning paradigms, like reward dependent tasks. Therefore, we evaluated cell proliferation, neurogenesis, astrogliogenesis, survival and neuronal maturation in the medial prefrontal cortex (mPFC) and the hippocampus (HIPP) during learning an operant conditioning task. This was performed by using endogenous markers of cell proliferation, and a bromodeoxiuridine (BrdU) injection schedule in two different phases of learning. Learning an operant conditioning is divided in two phases: a first phase when animals were considered incompletely trained (IT, animals that were learning the task) when they performed between 50% and 65% of the responses, and a second phase when animals were considered trained (Tr, animals that completely learned the task) when they reached 100% of the responses with a latency time lower than 5 seconds. We found that learning an operant conditioning task promoted cell proliferation in both phases of learning in the mPFC and HIPP. Additionally, the results presented showed that astrogliogenesis was induced in the medial prefrontal cortex (mPFC) in both phases, however, the first phase promoted survival of these new born astrocytes. On the other hand, an increased number of new born immature neurons was observed in the HIPP only in the first phase of learning, whereas, decreased values were observed in the second phase. Finally, we found that neuronal maturation was induced only during the first phase. This study shows for the first time that learning a reward-dependent task, like the operant conditioning, promotes neurogenesis, astrogliogenesis, survival and neuronal maturation depending on the learning phase in the mPFC-HIPP circuit.

## Introduction

Learning a task implies remodeling of neural circuits in the brain, these changes could be achieved by synaptic plasticity events as well as neurogenesis [1]. The operant conditioning task is one of the most important learning paradigms used in rodents for studying goal directed behaviors. This paradigm is guided by its consequences, for example, an animal that must press a lever to receive food as a reward. In rats, two of the areas involved in learning an operant conditioning task are the medial Prefrontal Cortex (mPFC) and the Hippocampus (HIPP). In previous reports by our group, we showed that in both areas while animals were acquiring the task, there was higher plasticity and activation compared to those animals that learned the task [2]–[4]. The dentate gyrus (DG) of the HIPP is one of two areas where adult neurogenesis takes place through adulthood and it is where this phenomenon has been associated to learning and memory [5], nevertheless, most of the research performed is related to spatial learning and memory tasks. New neurons through maturation process have changes in their membrane capacitance, type of inputs, synaptic connectivity and susceptibility for the induction of long term potentiation (LTP) [6]–[8]. In addition, learning not only influences the production of cells and the fate of these new cells [9]–[11], but also increases survival of cells that were born before training and, thereafter were subject to a selective process that allow some cells to live while others were eliminated [12]. Moreover, if the number of adult-born dentate granule cells at an immature stage is transiently reduced, learning impairments are generated [13]. On the other hand, the presence of neurogenesis in the cortex is still controversial, as some researchers found new neurons in primates and rats [14]–[16], whereas, other researchers reported the absence of neurogenesis trough adulthood [17], [18]. Astrocytes are key players in the formation and maturation of synapses, synaptic plasticity and LTP [19]–[22]. Therefore, it is imperative to know if in the mPFC there is astrogliogenesis associated to learning. Most of the research performed so far has been related to voluntary exercise, environmental enrichment and drug abuse, being unknown if this occurs among different learning paradigms. The aim herein was to study if learning an operant conditioning task promotes cellular proliferation in the mPFC-HIPP circuit, if it is associated to the degree of acquisition of the task and to identify the phenotype of these new cells. These results would bring better insights into the mechanisms of circuit modification during learning an operant conditioning task.

## Results

### Behavioral results

**Figure 1:**
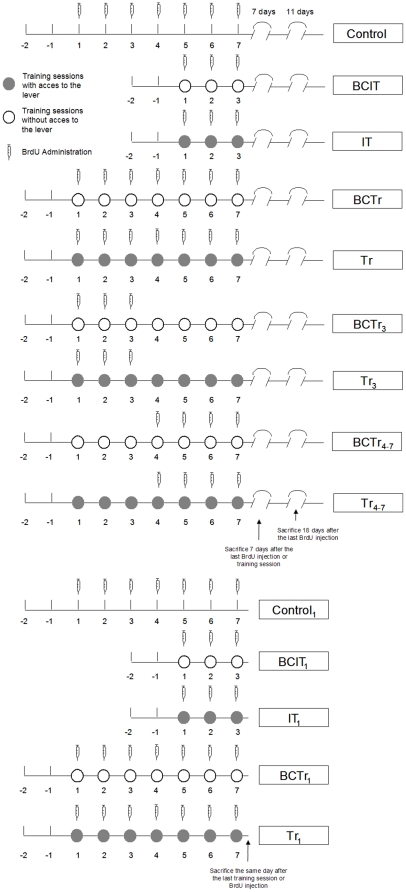
Experimental design and behavioral procedures. Syringes indicates BrdU injection in experimental groups. Animals from Control, ALC, IT, BCIT, Tr_3_ BCTr_3_, Tr_4-7,_ BCTr_4-7_, Tr and BCTr groups, were synchronized to be sacrificed as follows: seven days (for BrdU/DCX-IR and BrdU/GFAP-IR cell quantification), eighteen days (for BrdU/NeuN-IR cells quantification), or the same day after the last BrdU injection (BrdU/GFAP-IR cell quantification). For PCNA-IR cells quantification animals were sacrificed the same day after the last behavioral testing.

To study if learning a goal directed behavior could induce cell proliferation, neurogenesis, astrogliogenesis and neuronal maturation in the mPFC-HIPP circuit, we trained two groups of animals in an operant conditioning task. Animals were trained, injected and sacrificed in a schedule shown in Figure 1. For quantification of PCNA-IR cells, a group of animals were sacrificed in the same day or seven days after the last training session (Figure 1). On the other hand, for detection of BrdU/DCX-IR and BrdU/GFAP-IR cells, a group of animals was sacrificed 7 days after the last BrdU injection, whereas, another group of animals were sacrificed after the last BrdU injection for BrdU/GFAP-IR cells detection (Figure 1). Thereafter, another group of animals was sacrificed 18 days after the last BrdU injection (BrdU/NeuN-IR) (Figure 1). Animals from Tr group reached in the first session an average of 28.8%±2 of the responses (Table 1) with a mean latency time of 43%±5.3 seconds (Table 1), whereas, animals from IT group performed 31.2%±2.7 of the responses (Table 1) with an average latency time of 40.3%±4.5 seconds (Table 1). In the third session, animals from IT and Tr groups performed 61.8%±5.9 and 62.4%±4.7 of the responses, respectively (Table 1). Tr and IT groups also showed decreased latencies in the third session, where values reached an average of 27.8±3.2 and 25.7±2.9 seconds, respectively (Table 1). Animals that were completely trained performed 100% of the responses with a mean latency time of 3.9±0.63 seconds in the fifth session, they continued in this condition until the seventh session, when the latency time was 3.2±0.46 seconds (Table 1). Statistical analysis showed no significant differences in the mean latency time and percentage of responses between Tr and IT groups in the first, third an seventh session (Table 1).

**Table 1:** Operant conditioning task behavioral data.

	1° Session		3° Session		7° Session	
Group	% of Responses	Latency Time	% of Responses	Latency Time	% of Responses	Latency Time
Incompletely Trained (IT)	31.2±2.7	40.3±4.5	61.8±5.9	27.8±3.2	-	-
Trained (Tr)	28.8±2	43±5.3	62.4±4.7	25.7±2.9	100	3.2±0.4

Percentage of responses is expressed as the mean ± sem of correct responses in a 25 trials training session. Latency time is expressed as the mean ± sem of the time that elapses between lever presentation and lever pressing.

### Astrogliogenesis and cell proliferation in the mPFC

**Figure 2:**
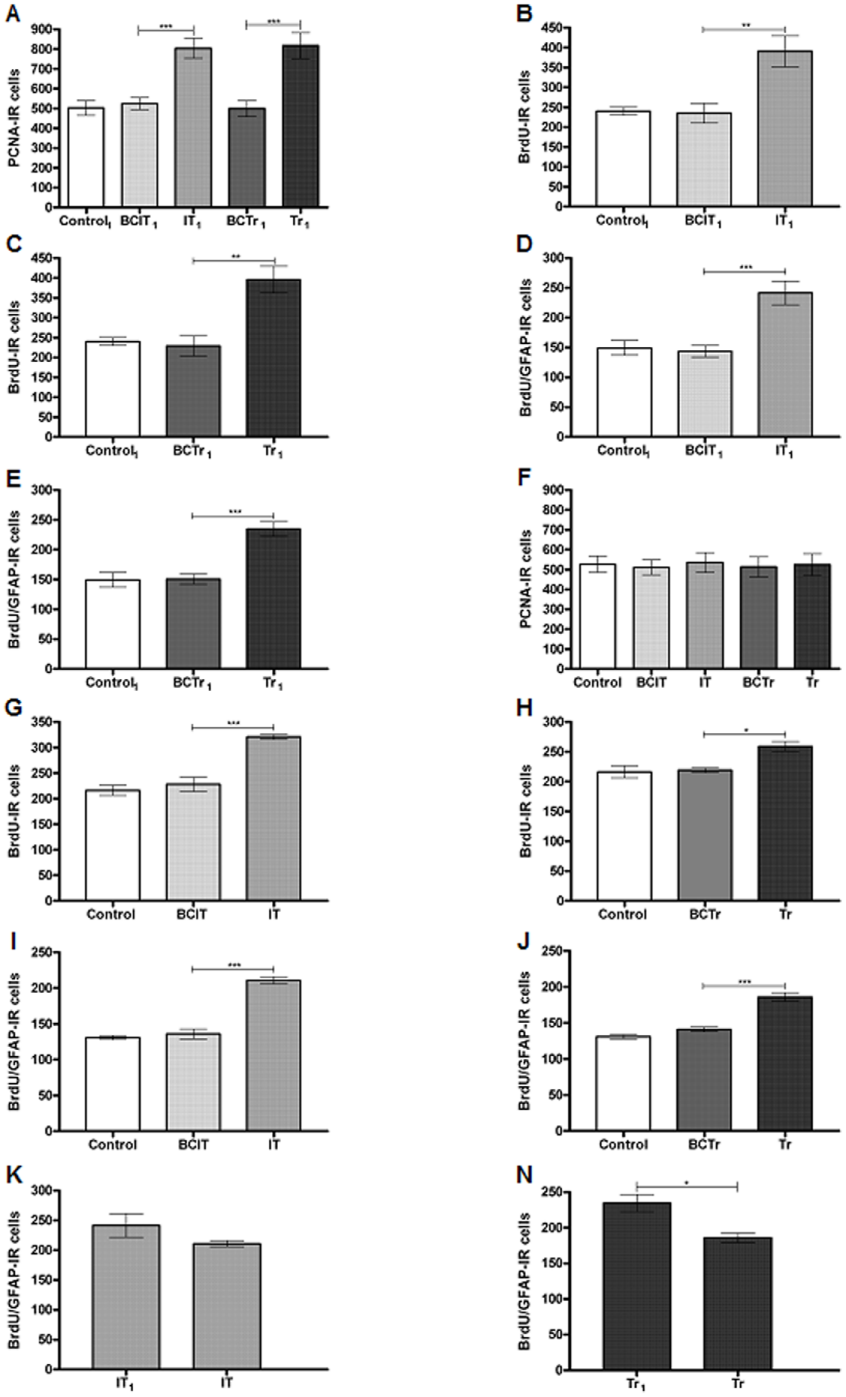
Cell proliferation and astrogliogenesisin the mPFC due to learning. PCNA-IR in the mPFC are expressed as the mean ± sem (panel A and panel F). BrdU-IR and BrdU/GFAP-IR cells in the mPFC from animals sacrificed the same day after the last BrdU injection are expressed as the mean ± sem (panel B-E). BrdU-IR and BrdU/GFAP-IR cells in the mPFC from animals sacrificed one week after the last BrdU injection are expressed as the mean ± sem (panel G-J). BrdU/GFAP-IR cells from IT_1_, IT, Tr and Tr_1_ groups are are expressed as the mean ± sem (panel K-N). Control_1_ (n = 10); BCIT_1_, Box Control of IT_1_ (n = 10); IT_1_ (n = 10); BCTr_1_, Box Control of Tr_1_ (n = 10), Tr_1_ (n = 10), Control (n = 10); BCIT, Box Control of IT (n = 10); IT (n = 10); BCTr, Box Control of Tr (n = 10), Tr (n = 10). *p<0.05,**p<0.01,***p<0.001. One way ANOVA followed by Tukey's post hoc test.

For studying cell proliferation in the mPFC, we analyzed the number of PCNA-IR cells when animals were in the first phase of learning (IT group) and when they were in the second phase of learning (Tr group). Quantification of PCNA-IR cells was performed in two different times: the same day after the last training session and 7 days after the last training session. All differences between group means were evaluated by one way ANOVA followed by post hoc Tukey's Multiple Comparisons Test for group comparison. A significant difference was found between the mean number of PCNA-IR cell of the experimental groups that were sacrificed the same day [F (4, 25, 13.85), p<0,001] (Figure 2A). A comparison carried out between IT_1_ and BCIT_1_ groups showed a significant increment in IT_1_ group [IT_1_ = 803.9±50.6, BCIT_1_ = 524.3±30.4; p<0.01] (Figure 2A). When Tr_1_ and BCTr_1_ groups were contrasted, there was an increment in the levels of PCNA-IR cells in Tr_1_ group [Tr_1_ = 817.7±65.6, BCTr_1_ = 499.9±40.7; p<0.001] (Figure 2A), whereas, no significant difference was found between Tr_1_ and IT_1_ groups [IT_1_ = 803.9±50.6, Tr_1_ = 817.7±65.6; p>0.05] (Figure 2A). In a second set of experiments, we analyzed BrdU incorporation in the mPFC in IT and Tr groups in two different times: the same day after the last training session and 7 days after the last training session. Learning the operant conditioning induced a difference in the mean number of BrdU-IR cells between experimental groups that were sacrificed the same day [F (4, 25, 11.97, p<0.001]. An increase in the number of BrdU-IR cells was found when IT_1_ and BCIT_1_ groups were compared [IT_1_ = 390.6±38.9, BCIT_1_ = 235±24.8; p<0.001] (Figure 2B). Also, an increment in Tr_1_ group was found with respect to BCTr_1_ [Tr_1_ = 395.8±33.8, BCTr_1_ = 229±25.6; p<0.001] (Figure 2C). Since a difference was found in the number of BrdU-IR cells, we proceeded to identify if these new born cells were glia or neurons. We used double labeling with: the glial fibrillary acidic protein (GFAP) as a marker for astrocytes, doublecortin (DCX) as a marker for immature neurons and neuron-specific nuclear protein (NeuN) as a marker for mature neurons. BrdU-IR cells from all groups were subjected to phenotypic analysis with DCX and NeuN. This quantification revealed the absence of immature or mature neurons in the mPFC. Nevertheless, we found that part of these new born cells were astrocytes. In fact, a difference among the mean value of BrdU/GFAP-IR cells was found between groups [F (4, 25, 16.89), p<0,001]. Comparison between IT_1_ and BCIT_1_ groups showed an increment in the number of BrdU/GFAP-IR cells in IT_1_ group [IT_1_ = 241.6±19.7, BCIT_1_ = 143.8±9.8; p<0.001] (Figure 2D). In addition, Tr_1_ group also showed augmented levels of BrdU/GFAP-IR cells with respect to BCTr_1_ [Tr_1_ = 234.6±12.1, BCTr_1_ = 150.7±8.7; p<0.001] (Figure 2E).

Statistical analysis between experimental groups of animals that were sacrificed 7 days after the last training session resulted in the absence of differences among means of these groups [F (4, 25, 0.4596, p = 0,9418] (Figure 2F). For animals that were sacrificed one week later after the last BrdU injection, a difference on the mean value of BrdU-IR cells between groups was observed [F (4, 25, 23.98, p<0,001]. Then, we found an increased number of BrdU-IR cells in the IT group compared to BCIT [IT = 321.2±5.2, BCIT = 228,8±13.2; p<0.001] (Figure 2G). Also, the Tr group showed augmented levels of BrdU-IR cells with respect to the BCTr group [Tr = 259.6±8.3, BCTr = 219.4±4.6; p<0.05] (Figure 2H). As it was found for animals that were sacrificed the same day, we were unable to find BrdU/DCX-IR or BrdU/NeuN-IR cells. Nevertheless, we found that part of these cells were BrdU/GFAP-IR and statistical analysis showed differences among groups [F (4, 25, 43.08, p<0,001]. Interestingly, a comparison carried out between IT and BCIT groups revealed a higher number of BrdU/GFAP-IR cells in the IT group[IT = 210.9±5.9, BCIT = 136.7±13.2; p<0.001] (Figure 2I). Later on, the comparison between Tr vs BCTr groups showed that there was an increment in the number of BrdU/GFAP-IR cells in the Tr group [Tr = 186.1±6.5, BCTr = 141±3.2; p<0.001] (Figure 2J). To elucidate if learning an operant conditioning task promoted survival of the astrocytes that were generated during learning, we compared IT and Tr groups from animals that were sacrificed the same day and seven days after the last BrdU injection. Comparison between IT_1_ and IT groups showed that learning promoted survival of the BrdU/GFAP-IR cells generated during task acquisition [IT_1_ = 241.6±19.7; IT = 210.9±5.9; p>0.05] (Figure 2K). On the contrary, the Tr group showed lower levels of BrdU/GFAP-IR cells contrasted with animals from the Tr_1_ group [Tr_1_ = 234.6±12.1,Tr = 186.1±6.5; p<0.05] (Figure 2N). To discard that food deprivation had a negative influence on new born cell production in the mPFC, we compared the number of BrdU-IR in animals that were food deprived with animals that were *ad libitum* during training sessions. This comparison resulted in the lack of differences between the Control and ALC groups [Control = 216.3±10; ALC = 207.5±9.4; p>0.05]. Moreover, food deprivation previous to training sessions showed no detrimental effects over the new born cell production rate in the mPFC, when it was compared with animals that were *ad libitum* during the same time period [CP = 230,7±15,8; CPF = 229,6±12,5; p>0,05].

### Neurogenesis and neuronal maturation through learning in the HIPP

**Figure 3:**
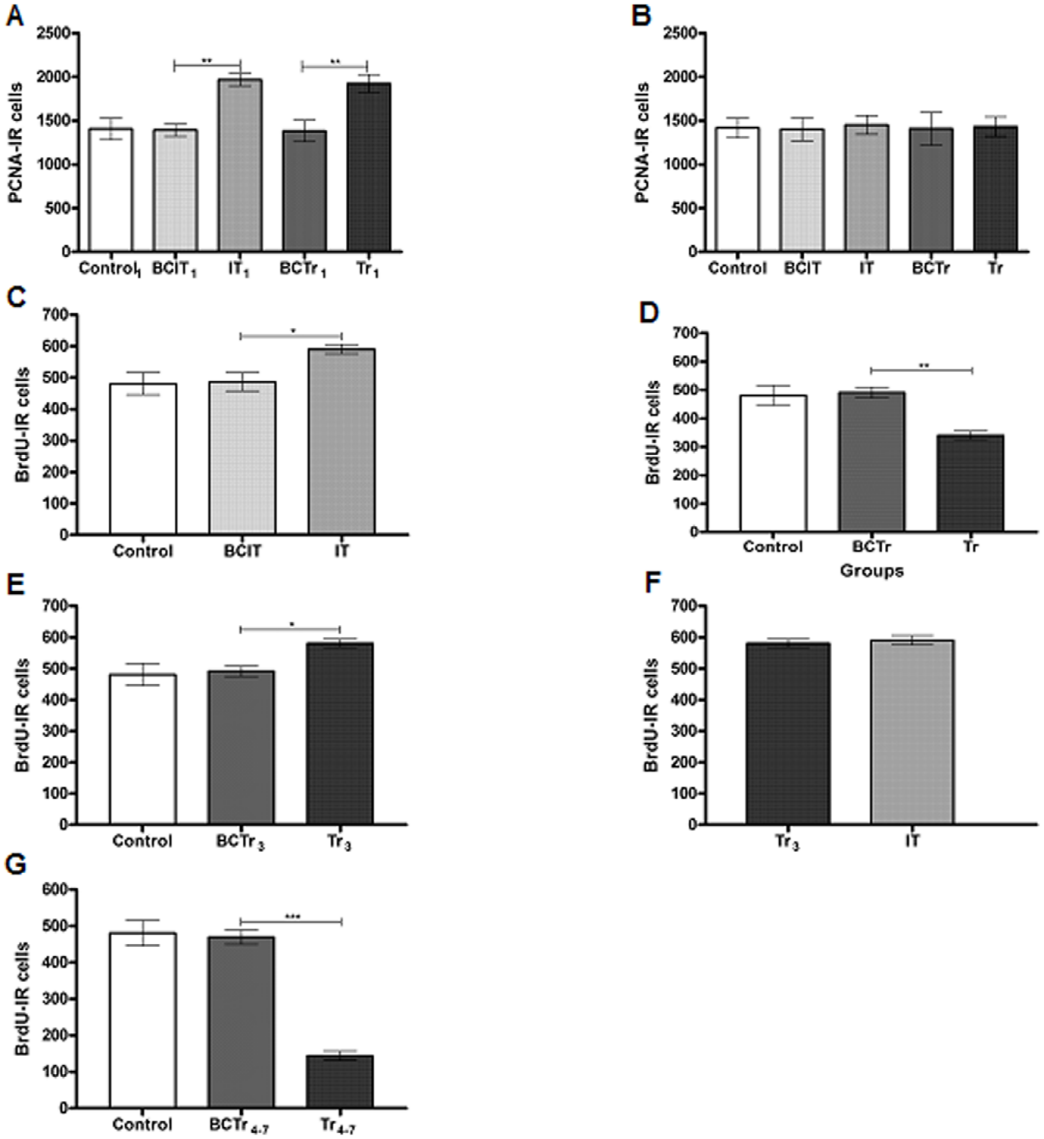
Differential cell proliferation and BrdU incorporation in the DG of the HIPP during learning. PCNA-IR in the HIPP are expressed as the mean ± sem (panel A-B). BrdU-IR -IR cells among experimental groups in the HIPP are expressed as the mean ± sem (Panel C-G).. Control_1_ (n = 10); BCIT_1_, Box Control of IT_1_ (n = 10); IT_1_ (n = 10); BCTr_1_, Box Control of Tr_1_ (n = 10), Tr_1_ (n = 10); Control (n = 10); BCIT, Box Control of IT (n = 10); IT (n = 10); BCTr, Box Control of Tr (n = 10), Tr (n = 10); BCTr_3_, Box Control of Tr_3_ (n = 10), Tr_3_ (n = 10), BCTr_4-7_, Box Control of Tr_4-7_ (n = 10), Tr4-7 (n = 10). *p<0.05,**p<0.01,***p<0.001. One way ANOVA followed by Tukey's post hoc test.

Cell proliferation in the HIPP was evaluated by quantification of PCNA-IR cells in two different times: the same day or seven days after the last experimental procedure. The number of PCNA-IR cells of animals that were sacrificed the same day showed differences among experimental groups [F (4, 25, 9.156, p<0,001]. Comparison between IT_1_ and BCIT_1_ groups resulted in an increment towards IT_1_ group [IT_1_ = 1968.5±70.8, BCIT_1_ = 1391.2±70.7; p<0.01] (Figure 3A). Similarly, Tr_1_ group showed augmented levels of PCNA-IR cells with respect to BCTr_1_ group [Tr_1_ = 817.7±65.6, BCTr_1_ = 499.9±40.7; p<0.001] (Figure 3A). On the contrary, there was no statistical difference between IT_1_ and Tr_1_ groups [IT_1_ = 1968.5±70.8, Tr_1_ = 817.7±65.6; p>0.05] (Figure 3A). In animals that were sacrificed one week after the last experimental procedure, the number of PCNA-IR cells did not change between groups [F (4, 25, 0.022, p = 0,986] (Figure 3B). In a first set of experiments, we evaluated the number of Brdu-IR cells in the DG; this quantification showed a fluctuation among the mean values of the groups [F (4, 25, 16.39, p<0.001]. Comparison between BCIT and IT groups showed that the first phase of learning induced an increment in the number of BrdU-IR [IT = 590.6±14.9, BCIT = 486.4±20.6; p<0.05] (Figure 3C). When Tr and BCTr groups were compared, we found that when the task was learned, the number of BrdU-IR cells were significantly decreased [Tr = 340.5±15.21; BCTr = 490.9±18.9; p<0.001] (Figure 3D). Using a different injection schedule for Tr animals, i.e. animals were injected during the first three sessions (Tr_3_ group) or the last four sessions (Tr_4-7_); we studied if learning promotes survival of the cell generated through acquisition and in which phase of learning occurred. The number of BrdU-IR cells in Tr_3_ group were augmented compared to the respective box control [Tr_3_ = 580.9±13.8; BCTr_3_ = 491.2±16.1; p<0.001] (Figure 3E). Additionally, comparison between Tr_3_ and IT resulted in no differences between these groups [Tr_3_ = 580.9±13.8; IT = 590.6±14.9; p>0.05] (Figure 3F). On the other hand, the Tr_4-7_ group showed decreased levels of BrdU-IR cells compared with BCTr_4-7_ [Tr_4-7_ = 144.7±12.4; BCTr_4-7_ = 469.7±19.7; p<0.001] (Figure 3G).

**Figure 4:**
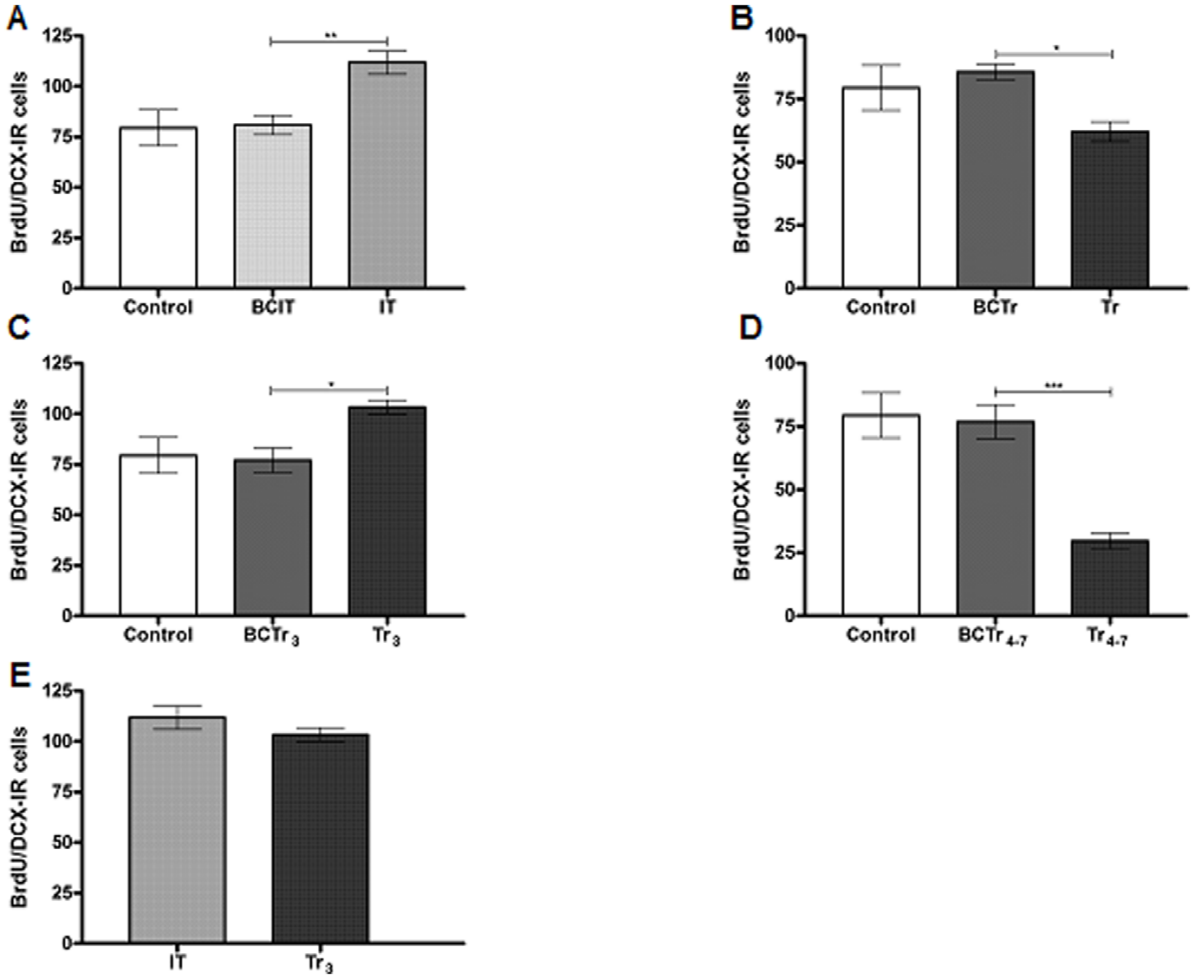
New born immature neurons in the DG of the HIPP during learning. BrdU/DCX-IR cells are expressed as the mean ± sem (Panel A-E). Control (n = 10); BCIT, Box Control of IT (n = 10); IT (n = 10); BCTr, Box Control of Tr (n = 10), Tr (n = 10), BCTr_3_, Box Control of Tr_3_ (n = 10), Tr_3_ (n = 10), BCTr_4-7_, Box Control of Tr_4-7_ (n = 10), Tr_4-7_ (n = 10). *p<0.05,**p<0.01,***p<0.001. One way ANOVA followed by Tukey's post hoc test.

**Figure 5:**
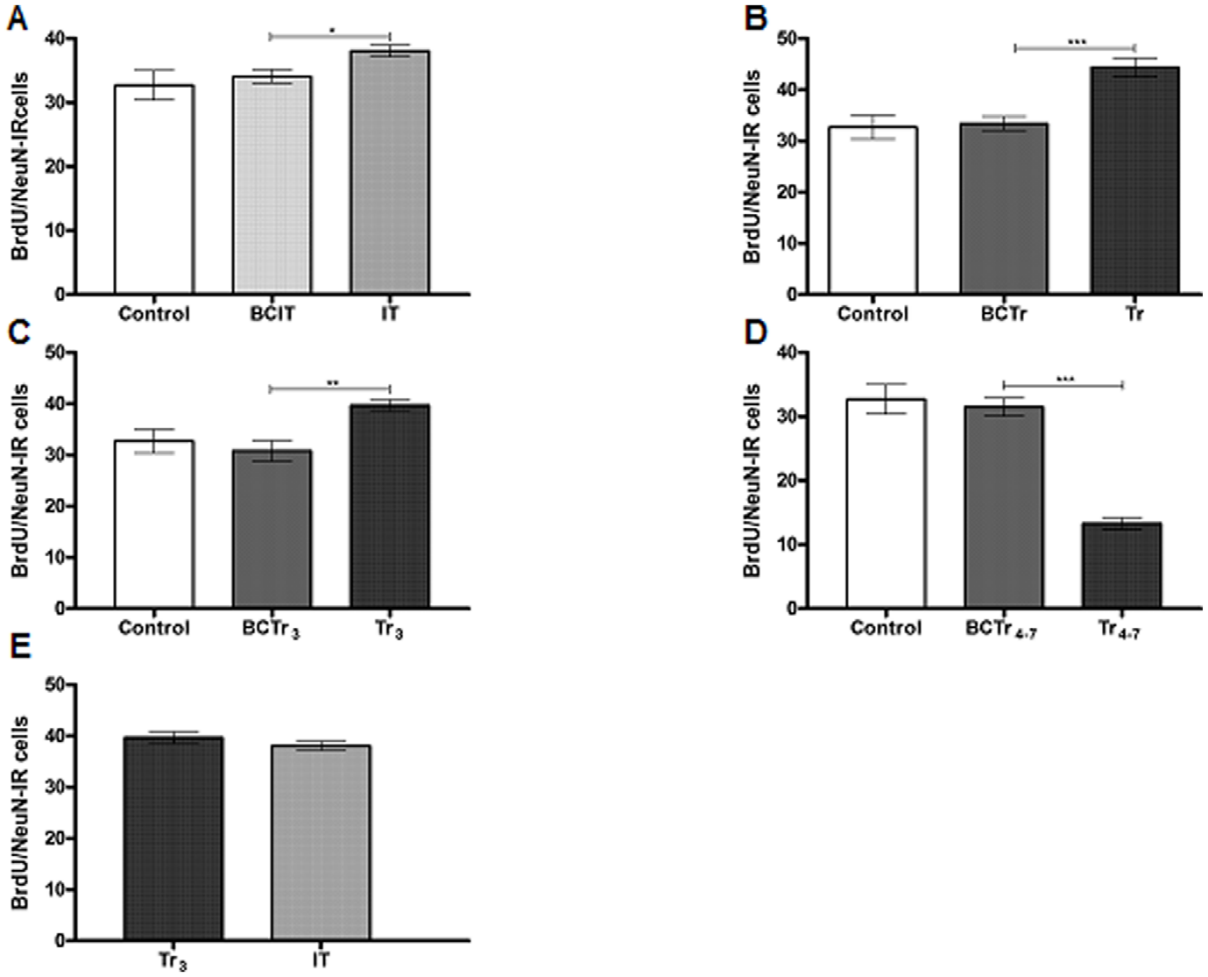
Adult born mature neurons in the DG of the HIPP during learning. BrdU/DCX-IR cells are expressed as the mean ± sem (Panel A-E). Control (n = 10); BCIT, Box Control of IT (n = 10); IT (n = 10); BCTr, Box Control of Tr (n = 10), Tr (n = 10), BCTr_3_, Box Control of Tr_3_ (n = 10), Tr_3_ (n = 10), BCTr_4-7_, Box Control of Tr_4-7_ (n = 10), Tr_4-7_ (n = 10). *p<0.05,**p<0.01,***p<0.001. One way ANOVA followed by Tukey's post hoc test.

**Figure 6:**
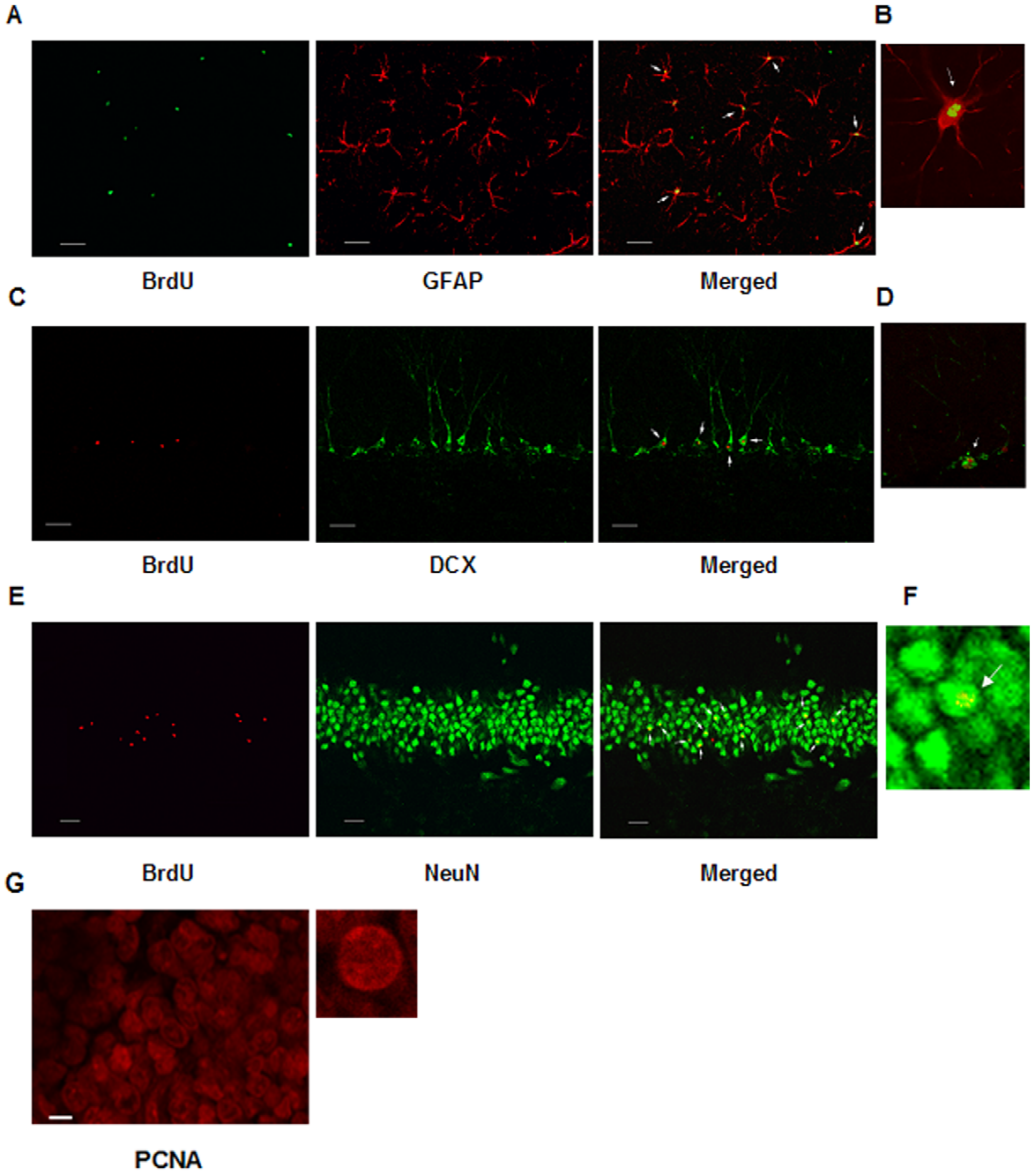
Illustration of phenotypic analysis of new born cells in the mPFC and HIPP. Double positive cells for BrdU (green) and GFAP (red) in the prelimbic region of mPFC from animals of the IT group (panel A and B). BrdU-IR cells (red) stained with DCX (green) in the granule cell layer of the HIPP (panel C and D) from animals of Tr_3_ group. New born mature neuron marked with NeuN (green) and BrdU (red) in the DG of the HIPP (panel E and F) from animals of Tr group. Arrowhead in each image points to a double positive cell for BrdU/NeuN, BrdU/DCX and BrdU/GFAP. PCNA-IR in the in the prelimbic region of mPFC from animals of the Tr group (panel G). Bar scales for A, C and E panels indicate 20 µm. Bar scale for G panel indicates 10 µm.

Quantification of BrdU/DCX-IR cells revealed significant differences among experimental groups [F (4, 25, 11.85, p<0,001]. The IT group showed a greater number of BrdU/DCX-IR cells than the BCIT group, [IT: 111.87±5.6; BCIT: 80.9±4.8; p<0.01] (Figure 4A). In addition, animals which completely learned the task showed lower levels of BrdU/DCX-IR compared to their respective box control [Tr: 62.1±3.9; BCTr: 85.7±3.1; p<0,05] (Figure 4B). Thereafter, quantification of BrdU/DCX-IR cells in animals from the Tr_3_ group showed an increment with respect to the BCTr_3_ group [Tr_3_:103.4±6.9; BCTr_3_: 77.1±6.1; p<0,05] (Figure 4C). Also, a decrease in the Tr_4-7_ group in the mean value of BrdU/DCX-IR cells was found with respect to the respective box control [Tr_4-7_ = 29.7±3.2; BCTr_4-7_ = 76.9±6.8; p<0.001] (Figure 4D). A comparison carried out between IT and Tr_3_ groups revealed the absence of differences [IT: 111.87±5.6; Tr_3_:103.4±6.9; p>0.05] (Figure 4E). Next, we examined the number of BrdU/NeuN-IR cells in the two phases of acquisition of an operant conditioning task. Statistical analysis of the number of BrdU/NeuN-IR cells between experimental groups showed differences among mean values [F (4, 25, 8.948), p<0.001]. Here, we found that the IT group had a higher number of BrdU/NeuN-IR cells with respect to the BCIT group [IT: 38.1±0.9; BCIT: 34.1±1.05; p<0.05] (Figure 5A), whereas, the Tr group also showed a significant increment compared with the BCTr group [Tr: 44.4±1.8; BCTr: 33.4±1.4; p<0.001] (Figure 5B). Besides, augmented levels of BrdU/NeuN-IR cells was found between Tr_3_ vs BCTr_3_ groups [Tr_3_:39.7±1.12; BCTr_3_: 30.8±6.1; p<0.01] (Figure 5C). Also, there was a statistically significant difference between Tr_4-7_ and BCTr_4-7_ groups [Tr_4-7_:13.3±0.9; BCTr_4-7_: 31.5±1.4; p<0.001] (Figure 5D). Additionally, a comparison carried out between IT and Tr_3_ groups resulted in the absence of differences [IT: 34.1±1.05; Tr_3_:39.7±1.12; p>0.05] (Figure 5E). Afterwards, we found that food deprivation during training sessions had no detrimental effects over basal cell production [Control:480,6±34,9; ALC:487,8±15,6; p>0,05]. Food deprivation previous to the experimental procedures showed no side effects over the new born cell rate in the HIPP [CP:230.7±15.8; CPF:229.6±12.5; p>0.05]. Finally, illustrations of what has been counted as Brdu/GFAP-IR cells, BrdU/DCX-IR cells, BrdU/NeuN-IR cells and PCNA-IR cells are presented in Figure 6.

## Discussion

Herein, it was found that astrogliogenesis, neurogenesis and neuronal maturation occurred in the mPFC-HIPP circuit during learning an operant conditioning task. These results support the hypothesis that neural circuits associated with learning could be modified by addition and maturation of new born neurons in the HIPP and by addition of new born astrocytes in the mPFC. We found also that learning promotes cell proliferation in the mPFC and that process is independent of the acquisition phase. Moreover, the mean number of PCNA-IR cells diminished to control levels after seven days of the last training session of animals that were completely trained or incompletely trained. Although learning promoted cell proliferation in the mPFC during learning, it seems that the stimulus necessary to maintain proliferation finished once the animal trained. Then, BrdU injection experiments showed that learning promoted cell survival of those cells generated during task acquisition. Phenotypic analysis of BrdU-IR cells generated during learning together with DCX and NeuN markers showed the absence of adult neurogenesis in the mPFC. Such results are in agreement with previous findings [17], [18], [23], [24]. Here, we showed that learning promotes astrogliogenesis independently of the degree of task acquisition. However, survival of these new born astrocytes was affected by the phase of learning. The fate of these cells was deeply associated to the degree of task acquisition: animals that completely learned the task showed a lower survival probability compared to animals that were learning the task, indicating that the first phase of learning is critical to this process. Modulation of astrogliogenesis has been observed in other circumstances, previous works have shown that voluntary exercise promotes astrogliogenesis in different regions of the cortex [25]. Methamphetamine self-administration also increased astroglionesis, nevertheless, the mechanisms underlying are different [26]. One possible explanation is that the dopamine released due to reward reception and prediction could be modulating astrogliogenesis [26]–[28]. This hypothesis emerges in part by results of our group where we found that dopamine was higher when animals were learning than when the task was completely learned (data not shown). However, the mechanisms in learning by which astrogliogenesis occurs are poorly understood. Since astrocytes are organized in networks to regulate plasticity, learning and function of neural circuits [29], [30], we do not discard a possible role in circuit formation inside the mPFC of these new born astrocytes during learning the task. Here, our results show that learning an operant conditioning task induced cell proliferation, astrogliogenesis and promoted astrocyte survival in the mPFC. Since all these events are deeply related to the acquisition phase, these results agree with previous findings by our group showing that while animals were learning the task, circuit modification was high [2], [3], [4].

Previous studies by different groups [2], [4], [31]–[33] showed that the HIPP is involved in learning an operant conditioning task. The DG of the HIPP is one of the places in the brain where neurogenesis takes place and there is plenty of evidence that connects this phenomena to learning, memory and cognition [34], [35]. Nevertheless, there is no information whether a reward dependent task could induce neurogenesis. Previous reports showed that enrichment and learning could induce cell proliferation within the HIPP [36], [37]. In this manuscript, we showed that cell proliferation was augmented in the first and second phase of learning an operant conditioning task. Actually, when animals were sacrificed one week after the last training session revealed no differences in cell proliferation, meaning that cell proliferation occurred and was induced only during learning without distinction of the learning phase.

New born immature neurons have enhanced synaptic plasticity, lower threshold to induce LTP and their activity rate is critical for being integrated in fully functional networks [7], [38]–[46]. Integration and activation of new born neurons into functional circuits are critical to learning and memory [47], [48]. Here, we found that learning an operant conditioning task differentially promotes neurogenesis and survival of new neurons in the HIPP. Nevertheless, this occurred during the first phase of learning while animals were learning the task. It was in this period when we observed that neurons generated in this phase has higher probability of survival. Although cell survival by learning has been previously reported in other paradigms [49], this is the first evidence that showed learning-induced survival in a reward dependent task. On the contrary, animals that were injected between the 4^th^ and 7^th^ sessions showed a strong decrement in the number of immature neurons when the task was learned. One possible explanation is that part of these cells were actively eliminated depending on the phase of learning, as was previously observed in a spatial task [11]. Whereas, another interpretation of these results could be that generation of new born cells was reduced. For animals completely trained that were injected during all training sessions, we observed that the number of new born immature neurons was considerably reduced. Taking together the results presented here, it seems that the first phase of learning promoted cell survival, but it is not promoted in the second phase of learning. Herein, we found that there was no correlation between neurogenesis and learning, this discrepancy with other reports [36], [10] could be explained due to the different role of the HIPP in a reward dependent task and a spatial learning. This mechanism of learning-induced survival in different phases of learning has been reported in the hippocampus in a water maze task [50], [10]. Considering that this new born immature neurons are functional and codify information even in the early stages before maturation [51], our results suggest that this could be a common mechanism of circuit formation in the hippocampus for different learning and memory tasks. Together with previous findings by our group [2], [4], we propose that all major circuit modification in the HIPP occurred during the first phase of learning an operant conditioning task. In another set of experiments to study the influence of the phase of learning over neuronal maturation, we showed that the first and second phase of the operant conditioning task had different effects. Further examination indicated that the first phase of learning provides the stimuli necessary to promote neuronal maturation of the newly born immature neurons. Learning has been proposed as a key player of maturation of new born neurons by inducing the expression of proneural genes and acceleration of synaptic inputs [52], [53]. Moreover, the number of BrdU-NeuN cells was increased in animals that were subjected to a spatial learning task [53]. We propose a similar mechanism promoted by learning an operant conditioning task. This could be part of late modifications in the HIPP for acquisition of the task and formation of the neural circuits involved in learning.

Our group showed for the first time that a reward dependent task differentially induces cell proliferation, cell survival, astrogliogenesis, neurogenesis and neuronal maturation in the mPFC-HIPP circuit. This differential regulation in the mPFC-HIPP circuit was dependent on the phase of learning, when the first phase promoted cell proliferation, neuronal maturation and survival of astrocytes and immature neurons generated in this phase. Although the second phase of learning induced cell proliferation and astrogliogenesis, most of cells generated in this phase had lower chances of survival in both structures of the brain. These results showed that addition and survival of new born cells in the mPFC-HIPP circuit is differentially regulated through acquisition of an operant conditioning task and that these events could be involved in the formation of the new circuits related to learning.

## Materials and Methods

### Experimental procedures

All experimental procedures were approved by ethics committee of IByME-CONICET (A2008) and were conducted according to the NIH Guide for Care and Use of Laboratory Animals.

### Animals

Two month old Male Long Evans rats (300–325 g) were provided by the IBYME-CONICET, maintained on a 12/12 h light/dark cycle with water ad libitum.

### Antibodies

The following primary and secondary antibodies were used: mouse monoclonal anti-glial fibrillary acidic protein (GFAP; 1∶200; Millipore, USA), rat monoclonal anti-BrdU (1∶100; AbCam, UK), goat polyclonal anti-doublecortin (DCX; 1∶200; Santa Cruz, USA), mouse monoclonal anti-neuronal nuclear protein (NeuN; 1∶100; Millipore, USA), mouse monoclonal anti- proliferating Cell Nuclear Antigen (PCNA; 1∶200; Millipore, USA), donkey anti-rat conjugated with Cy3 (Millipore, USA), donkey anti-goat conjugated with Cy5 (Millipore, USA), donkey anti-mouse conjugated with Cy5 (Millipore, USA).

### Operant conditioning task

All behavioral procedures were performed during the light cycle, the operant conditioning task trainings was performed in a standard operant chamber (MED associates inc, St. Albans, Vermont, USA) equipped with: an input (DIG 710/711) and output (DIG 720/721/722) card for data acquisition and processing, one automated retractable lever, white light house, context red light, white noise (random signal with a flat power spectral density) and automated feeder. All animals were single housed and handled every day for at least 12 days. Rats were then food restricted to maintain ∼80% of their ad libitum body weight for 3 days before training and throughout the experiments, followed by two days of habituation. Animals were first placed in the training room for 15 min followed by a 20 min of habituation in the operant chamber. In the habituation process, rats in the operant chamber were only exposed to context red light and white noise, and fed with 25 pellets (45 mg, BioServe) gave randomly by the automated feeder. One session of 25 trials was performed. A session begins with the lever retracted, the operant chamber white light on, and a red context light that remained on during the session. Each trial begins when the lever came out for 60 seconds and the operant chamber white light turns off, if the animal pressed the lever received a pellet of 45mg as a reward. The action of pressing the lever was considered as a correct response. When the trial finished, the white light turns on and the lever remained retracted for 20 seconds. If no response was performed during the trial, no reward was given. Incompletely Trained group (IT) criteria was to reach 50–65% of responses. Whereas for Trained (Tr), Trained_3_ (Tr_3_) and Trained_4-7_ (Tr_4-7_) groups criteria was to reach 100% of responses and a latency time below 5s for three consecutive sessions. Latency was calculated as the amount of time that elapses between presentation of the conditioned stimulus and occurrence of the lever pressing. If no response was performed, latency is the duration of the trial (in our case 60 sec). For Box Control of IT group (BCIT), Box control of Tr (BCTr), Box control of Tr_3_ (BCTr_3_) and Box control of Tr_4-7_ (BCTr_4-7_), sessions started with the house white light on, and a red context light on, then the white light turned off and the animal remained in the box with the lever retracted until IT ,Tr , Tr_3_ and Tr_4-7_ finished their training sessions. BCIT, BCTr, BCTr_3_ and BCTr_4-7_ spent the same amount of time in the operant chamber as the IT, Tr, Tr_3_ and Tr_4-7_ groups, respectively. To discard if food deprivation could affect the number of proliferating cells, two controls were included: Ad libitum Control (ALC, animals that were not food deprived during experimental procedures), deprived Control (CP, animals that were food deprived before and during experimental procedures) and non deprived control (CPF, animals that were not food deprived for seven days previous to experimental procedures). Experimental groups were as follows: Incompletely Trained (IT, n = 10), Box Control of IT (BCIT, n = 10), Trained (Tr, n = 10), Trained_3_ (Tr_3_, n = 10), Trained_4-7_ (Tr_4-7_, n = 10), Box Control of Tr (BCTr, n = 10), Control (Control, n = 10), Ad libitum Control (ALC, n = 10), deprived Control (CP, n = 10), Box control of Tr_3_ (BCTr_3_, n = 10), Box control of Tr_4-7_ (BCTr_4-7_, n = 10).

### BrdU Injections and tissue preparation

Rats were intraperitoneal injected with 50 mg/Kg of bromodeoxyuridine (Sigma, USA) two hours previously to behavioral procedures. For a detailed explanation of administration schedule and sacrifice see Figure 1. Animals were anesthetized with 100 mg/Kg of Ketamine and 20 mg/Kg of Xilazine and perfused transcardially with 200 ml of saline solution followed by 300 ml of 4% formaline/PBS solution with a peristaltic pump (Apema, Argentina). Then, brains were coronally sectioned with a vibratome at 50 µM trough the mPFC (3.7 to 2.2) and DG of the HIPP (−4,52 to 3,14) [54]. Slices were stored in 0.1% NaN3, PBS, 0.5% sacarose at 4°C.

### Immunocitochemistry

All sections were washed with TBS two times and incubated in Triton X-100 0.1% in TBS 1x (TBS-Tx 0.1%) buffer for 10′ at room temperature. To expose the BrdU incorporated into cells, free floating sections were incubated in 50% formamide/sodium citrate buffer 2x (SSC 2x) at 65°C for 90′. Then, sections were washed with sodium citrate buffer 2x and incubated in HCl 2N at 37°C for 20′ and later neutralized with borate buffer 0.1M ph = 8.5 for 10′. Next, two washes with TBS-Tx 0.1% were performed followed by a blocking solution of 3% of donkey serum (Millipore, USA) in TBS-Tx 0,1% for 45′to avoid unspecific binding. Afterwards, for double labeling experiments sections were simultaneously incubated with anti-BrdU for 48 h at 4°C and with one of the other primary antibodies (GFAP, DCX and NeuN) for 24 h at 4°. All primary antibodies were diluted in 1% of serum donkey in TBS. Then, free floating sections were sequentially incubated with the secondary antibodies conjugated with Cy3 and Cy5. Finally, sections were mounted in conventional slides with Vectashield (Vector Laboratories, USA). In all experiments lack of primary antibodies in the protocol of immunocitochemistry was considered as the negative control, resulting in the absence of signal.

### Confocal Imaging and Quantification

Quantification of mPFC and HIPP cell was performed by using the optical fractionator method in which the left and right hemisphere of every ninth section through the mPFC and HIPP was examined. Cells from each bregma region were summed and multiplied by nine to give the total number of cells. . This was performed from a series of photos taken at 200x, whereas, phenotypic analysis of BrdU-IR was performed in both hemispheres at 400x with a confocal microscope Olympus FV300 equipped with Ar 488 nm, HeNe 543 nm and HeNe 633nm lasers. For the HIPP, BrdU-IR were counted from sub-granule and granule cell layer, whereas, all the area of the mPFC was considered without distinction of sub-structures. Confocal analysis of phenotype was performed and restricted to the top 15 µm of the section where the penetration of all antibodies is reliable. Standards for BrdU/NeuN-IR assessment included 100% colocalization of BrdU-IR cell with NeuN, whereas, standards for BrdU/GFAP-IR cells assessment included 100% colocalization of BrdU-IR cell with GFAP. Colocalization of antibodies was assessed with the confocal system by analysis of adjacent z sections and orthogonal sectioning (x–y–z plane) through single z sections. Three-dimensional renderings were rotated, and colocalization was examined from x-, y-, and z-axes.

### Statistics

All the statistical analysis was performed using GraphPad Prism 4.00 (GraphPad Software, San Diego California USA). Values were expressed as means±SEM and compared using one way ANOVA and post hoc comparisons with Tukey's Multiple Comparisons Test, differences among experimental conditions were considered statistically significant when P<0.05.
